# Service user, carer and provider perspectives on integrated care for older people with frailty, and factors perceived to facilitate and hinder implementation: A systematic review and narrative synthesis

**DOI:** 10.1371/journal.pone.0216488

**Published:** 2019-05-13

**Authors:** Euan Sadler, Victoria Potterton, Ruth Anderson, Zarnie Khadjesari, Katie Sheehan, Farida Butt, Nick Sevdalis, Jane Sandall

**Affiliations:** 1 Centre for Implementation Science, Health Service and Population Research Department, Institute of Psychiatry, Psychology and Neuroscience, King’s College London, London, United Kingdom; 2 Faculty of Environmental and Life Sciences, University of Southampton, Southampton, United Kingdom; 3 Department of Population Health Sciences, School of Population Health & Environmental Sciences, King’s College London, London, United Kingdom; 4 School of Health Sciences, University of East Anglia, Norwich, United Kingdom; 5 Florence Nightingale Faculty of Nursing, Midwifery & Palliative Care, King’s College London, London, United Kingdom; 6 Department of Women and Children’s Health, School of Life Course Sciences, Faculty of Life Sciences & Medicine, King’s College London, St. Thomas' Hospital, London, United Kingdom; Nord University, NORWAY

## Abstract

**Introduction:**

Older people with frailty (OPF) can experience reduced quality of care and adverse outcomes due to poorly coordinated and fragmented care, making this patient population a key target group for integrated care. This systematic review explores service user, carer and provider perspectives on integrated care for OPF, and factors perceived to facilitate and hinder implementation, to draw out implications for policy, practice and research.

**Methods:**

Systematic review and narrative synthesis of qualitative studies identified from MEDLINE, CINAHL, PsycINFO and Social Sciences Citation Index, hand-searching of reference lists and citation tracking of included studies, and review of experts’ online profiles. Quality of included studies was appraised with The Critical Appraisal Skills Programme tool for qualitative research.

**Results:**

Eighteen studies were included in the synthesis. We identified four themes related to stakeholder perspectives on integrated care for OPF: different preferences for integrated care among service users, system and service organisation components, relational aspects of care and support, and stakeholder perceptions of outcomes. Service users and carers highlighted continuity of care with a professional they could trust, whereas providers emphasised improved coordination of care between providers in different care sectors as key strategies for integrated care. We identified three themes related to factors facilitating and hindering implementation: perceptions of the integrated care intervention and target population, service organisational factors and system level factors influencing implementation. Different stakeholder groups perceived the complexity of care needs of this patient population, difficulties with system navigation and access, and limited service user and carer involvement in care decisions as key factors hindering implementation. Providers mainly also highlighted other organisational and system factors perceived to facilitate and hinder implementation of integrated care for OPF.

**Conclusions:**

Similarities and differences in lay and professional stakeholder perspectives on integrated care for OPF and factors perceived to facilitate and hinder implementation were evident. Findings highlight the importance of addressing organisational and system level components of integrated care and factors influencing implementation for OPF. Greater attention needs to be placed on collaboratively involving service users, carers and providers to improve the co-design and implementation of integrated care programmes for this patient population.

## Introduction

Advances in healthcare and technology have resulted in older people living longer. However, despite gains in life expectancy, the likelihood of experiencing long term health conditions and associated disability increases with age [1]. Frailty is defined as an age-related reduction in reserve capacity resulting in an increased risk of sudden decline in health status, triggered by a minor stress, such as a fall or infection [2,3,4]. Older people with frailty (OPF) often have complex health and social care needs [5], experience multimorbidity [6] and are frequent users of health and social care services, with associated high costs [7]. Yet, OPF commonly experience reduced quality of care and adverse outcomes due to poorly coordinated and fragmented care, making this patient population a key target group for integrated care programmes [8,9,10].

Integrated care, broadly defined as ‘an organising principle for care delivery that aims to improve patient care and experience through improved coordination’ ([11]: p.3), is a widely proposed strategy to address variations in quality of care and increased costs arising from fragmented care systems [12]. However, current evidence for the benefits of integrated care for OPF is equivocal, with some studies reporting a benefit [13,14] and others no or insufficient evidence of a benefit [15,16] on patient and service outcomes. This may be due in part to different models and formulations of integrated care to support OPF and uncertainty as to the key components associated with positive outcomes [16,17].

Factors facilitating and hindering the implementation of integrated care in general [18], and more specifically in older adult populations [19,20], highlight a range of contextual factors operating on micro, meso and macro levels. For example, Kirst et al conducted a realist review of integrated care evaluations to identify mechanisms and contextual factors influencing the successful implementation of integrated care for older people with complex needs [20]. They identified two key ‘context-mechanism-outcome configurations’ focusing on 1) trusting multidisciplinary team (MDT) relationships; and 2) level of understanding and commitment to the integrated care intervention among providers. Contextual factors facilitating implementation included strong leadership shaping organisational cultural support for the intervention, team collaboration that was supportive, having sufficient time to develop the infrastructure for implementation, and flexibility of the implementation process [20].

However, little is known about lay and professional stakeholder understandings of integrated care for OPF, with any differences likely to affect the effectiveness and successful implementation of such models of care. This systematic review and narrative synthesis aimed to explore service user, informal carer (e.g. family members, hereafter referred to as carers) and provider perspectives on integrated care for OPF, and factors perceived to facilitate and hinder implementation, to draw out implications for policy, practice and future research.

## Methods

### Search strategy and study identification

We used different strategies to identify qualitative studies of stakeholder perspectives for this review, as recommended when searching for qualitative evidence from a range of sources [21]. MEDLINE, CINAHL, PsycINFO and Social Sciences Citation Index databases were searched from inception (i.e. date of origin in each database) to June 2017. The search strategy used MeSH and free text search terminology, combining search terms for type of stakeholder (e.g. service user, frail older adult, carer or health and social care provider) with terms for integrated care (e.g. integrated care, integrated care pathway) or type of integrated care model known to the researchers (e.g. Buurtzorg model), and qualitative research, implementation and related terms, which were adapted for each database (see S1 File for MEDLINE search). Studies identified were exported into EndNote X8 and titles and abstracts were first screened by two reviewers independently (VP, RA). This was followed by full text screening by three reviewers independently (VP, RA, ES). Two authors (VP, RA) also hand-searched reference lists, used citation tracking of included studies in Google Scholar, and reviewed online profiles of experts in the field to identify further eligible studies. Discrepancies were resolved by consensus (VP, RA, ES).

### Eligibility criteria

We included qualitative studies which examined service user, carer and/or provider (health and social care professionals) perspectives of integrated care for OPF, conducted in any geographical context, published in peer reviewed English language journals. Eligible service users were older people (aged ≥ 55 years) identified as frail or with complex health and social care needs and multimorbidity (frailty implied). We excluded studies using only quantitative methods (e.g. surveys, epidemiological research) and intervention studies. As OPF commonly experience multimorbidity [6], we also excluded qualitative studies focusing on the views of older people with single specific long term conditions (e.g. cancer, stroke). Additionally, we excluded commentaries, editorials, opinion pieces and conference abstracts.

### Data extraction and method of synthesis

A narrative synthesis was used to synthesise findings from included studies to produce a textual account of similarities and differences in stakeholder perspectives. We employed steps 2–4 of the Economic Social Research Council’s (ESRC) research methods framework guidance on conducting a narrative synthesis [22]. This guidance outlines four stages that can be conducted iteratively–develop a theory, develop preliminary synthesis of the findings of included studies, explore relationships in the data, and examine robustness (quality) of the synthesis. We did not use step one as this was not the focus of our review.

We used tabulation and thematic analysis to compare similarities and differences between lay and professional perspectives. Two authors (VP, RA) developed tables with relevant sub-headings, i.e. author/year/country; aim; sample; methods; theoretical perspective; and quality appraisal scores (see Table 1). Tables including themes for service user, carer and provider perspectives on integrated care for OPF, and factors perceived to facilitate and hinder implementation were constructed to compare the views of different stakeholder groups (see Tables 2 and 3 respectively). Following tabulation, three authors (VP, RA, ES) used the ‘one sheet of paper’ analysis approach [23], to visually display themes and related subthemes across different stakeholder perspectives and ascertain relationships between these. Final themes and related subthemes were determined by consensus (VP, RA, ES).

**Table 1:** Study characteristics and methodological quality of studies.

Author, Year, Country	Aim	Sample	Methods	Theoretical Perspective	Quality score (CASP)
Boudioni et al 2015 UK [26]	To explore service users’ and family carers’ perspectives of an integrated care service in London	5 service users with complex health conditions and 5 carers	Video stories analysed between researchers and service users, as part of an experience-based co-design evaluation	Principles of visual sociology	8.5
Hu (2014) UK [27]	To examine service users’ views of the impact of an integrated care service in England	100 older care service users surveyed, then 27 older adults aged ≥ 65 years with complex care needs selected	Mixed methods: survey then face-to-face interviews with sub-sample of participants	Not specified	5.5
Ballie et al (2014) UK [28]	To investigate service users’ and providers’ perspectives of care transitions in a vertically organised integrated healthcare system	17 providers, including range of acute (N = 8) and community care providers (N = 9) and 4 older adults with frailty aged ≥ 70 years	Qualitative case study using face-to-face interviews with key staff and patients, and focus groups with ward staff	Ritchie & Spencer’s (1994) five-stage framework analysis	8
Bone et al (2016) UK [29]	To explore views of service users and other key stakeholders to inform development of a short-term community-based integrated palliative and supportive care intervention for OPF	63 participants (healthcare providers, commissioners, voluntary sector representatives, carers, researchers) took part in stakeholder consultations; 42 participants (providers, carers, researchers) took part in survey; 8 frail older people aged ≥ 75 years and 9 carers	Expert stakeholder consultations and follow-up consensus surveys with providers, carers and researchers; focus groups with service users and carers	Not specified	10
Sheaff et al (2009) UK [30]	To elicit service users’, carers’ and providers’ perspectives on the impacts of different case management systems across 9 primary care trusts in England	Range of providers working in acute, primary, secondary and community care (N = 70); 72 older people aged ≥ 65 years with range of long term conditions, and 52 informal carers	Multiple case study evaluation design; face-to-face interviews, observations of meetings and analysis of key documents	Not specified	7
de Stampa et al (2009) Canada [31]	To examine incentives and barriers among GPs to take part in integrated health services networks (IHSNs) to enable integrated care for frail older adults in Montreal	61 GPs enrolled in an integrated care system for older adults, of which a random sample of 22 GPs actively or non-actively participating in IHSNs recruited	Initial mail survey, then subsample of GPs took part in face-to-face interviews	Not specified	7.5
Heckman et al (2013) Canada [32]	To identify providers’, service users’ and carers’ perspectives on improving integration care for frail seniors in Ontario	186 providers in primary, secondary and community care and 29 service users and carers	Secondary analysis of 20 focus group discussions	Not specified	7
Lafortune et al (2015) Canada [33]	To explore older adults’, carers’ and providers’ views on improving primary healthcare community services for older adults with long term conditions in an area of Ontario	Range of healthcare providers (N = 20); 28 service users aged ≥ 65 with experience of one or more services (e.g. chronic disease management, end of life care) and their informal carers	Focus groups with care providers (N = 4) and service users and carers (N = 3), and individual interview with informal carer	Not specified	7.5
McAiney et al (2017) Canada [34]	To examine service users’, carers’ and providers’ perceptions of the impact of an intensive geriatric service worker (IGSW) service in South Ontario	19 providers (IGSW program lead, case manager, nurses, geriatrician); 49 service users aged ≥ 65 with age-related conditions; 25 informal carers	Mixed methods design; initial patient satisfaction survey; purposive sample of all stakeholders took part in telephone interviews (N = 93)	Not specified	6.5
Spoorenberg et al (2015) The Netherlands [35]	To examine service users’ views of a community-based integrated care intervention based on the Chronic Care Model	23 older adults aged ≥ 75 years, mostly with frailty or complex needs, sampled from a trial	Face-to-face interviews 8–10 months after starting the intervention	Grounded theory approach	9
Janssen et al (2015) The Netherlands [36]	To examine providers’ views of organisational features facilitating implementation of community integrated care for older adults	12 providers (nurses, case managers, GP, nursing home manager, homecare worker, geriatrician)	Qualitative case study: face-to-face interviews, observations of team meetings, then focus groups to discuss findings	Nomological network of organisational empowerment (Peterson & Zimmerman 2004)	8.5
Metzelthin et al (2013) The Netherlands [37]	To examine service users’ and providers’ experiences of an interdisciplinary primary care model for OPF, and perceived barriers and enablers to implementation in south Holland	45 care providers (nurses, GPs, allied health providers); 194 service users aged ≥ 70 years scoring ≥ 5 on the Groningen Frailty Indicator. Participants recruited from 6 GP practices	Mixed methods process evaluation: quantitative log books and evaluation forms for all service users; interviews with subsample of 13 participants; focus groups (N = 4) and interviews (N = 12) with providers	Baranowski and Stables’ (2000) process evaluation model	7.5
Hjelm et al (2015) Sweden [38]	To explore service users’ experiences of case managers	13 older adults aged ≥ 75 years with ≥ 3 long term conditions who received the case management intervention	Focused ethnographic approach including observations of case manager practices and face-to-face interviews	Roper & Shapira’s (2000) framework for ethnographic analysis	8
Dunér et al (2011) Sweden [39]	To examine providers’ views of implementing a new continuum of care model for frail older people	26 providers (upper managers, nurses, allied health providers, social workers, case managers)	Repeat face-to-face interviews, as part of a trial of the intervention	Lipsky’s (1980) theory of street-level bureaucracy	6
Freij et al (2011) USA [40]	To examine older adults’ experiences of care coordination services in the New York City area	48 older adults aged ≥ 55 years (majority ≥ 75 years) from multi-ethnic backgrounds, targeting older adults with frailty	Face-to-face interviews (N = 25) and focus groups (N = 6)	Grounded theory approach	7.5
Keefe et al (2009) USA [41]	To examine primary care physicians’ and nurses’ views of implementing integrated care for frail older people, and the benefits of social worker integration in primary care teams	25 providers (13 physicians, 11 nurses, 1 nurse practitioner) working in primary care	Focus groups (N = 3) conducted at 2 primary care clinics	Grounded theory approach	5.5
Busetto et al (2017) Germany [42]	To explore providers’ views of implementing an integrated care model in a German hospital, as part of a comparative European project	15 MDT care providers (physicians, nurses, allied health providers, psychologists, social workers)	Face-to-face interviews	Wagner's (1998) Chronic Care Model, Grol and Wensing's (2004) Implementation Model, Realist evaluation approach	8.5
de Stampa et al (2013) Canada/France [43]	To understand the clinical collaboration process between primary care physicians (PCPs), case managers and geriatricians in two integrated care systems for frail older adults	46 care providers (35 PCPs, 7 case managers, 4 geriatricians)	Face-to-face interviews	Grounded theory approach	7.5

**Table 2:** Service user, carer and provider perspectives on integrated care for older people with frailty.

Themes and related sub-themes	Service users	Carers	Providers
**Different preferences for integrated care among service users**	[26,35,38]		
**System and service organisation components of integrated care for older people with frailty**			
Improved continuity and coordination of care, and multidisciplinary team working	[27–30,33–35,38,40]	[29,30,33,34]	[28–30,32–34,36,41–43]
Improved access and navigation of the health and care system for service users and carers	[27,29,30,32,33,38]	[30,32,34]	[32–34]
**Relational aspects of care and support as part of integrated care for older people with frailty**			
Quality and nature of service user-provider relationships	[27,30,35,37,38,40]	[30]	
Access to appropriate and timely carer support	[29,32]	[29,30,32,34]	[28,29,32]
**Stakeholder perceptions of outcomes of integrated care for older people with frailty**			
Improved service user and carer outcomes	[26,27,29,30,35,40]	[29]	[29]
Improved system and organisational processes and service outcomes	[28,30,32–35,38,40]	[28,30,33,34]	[28,30,32–34,36,37,41–43]

**Table 3:** Service user, carer and provider perceptions of factors facilitating and hindering implementation of integrated care for older people with frailty.

Themes and related sub-themes	Service users	Carers	Providers
**Perceptions of the integrated care intervention and target population**			
Provider views of the perceived value of the intervention			[28,31,37,39]
Complexity of care needs of the patient population	[29,32,33,35]		[29,32]
**Service organisational factors influencing implementation**			
Poor communication and the nature of collaborative working practices between providers	[33]	[33]	[28,30,31,33,36,39,43]
Level of engagement of managers, frontline staff and primary care physicians in the implementation process			[30–32,36,39,41,43]
**System level factors influencing implementation**			
Limited support for service users and carers to navigate and access the health and care system and availability of infrastructure to support and fund integrated care	[29,32,33]	[29,32,33]	[28,29,32,33,42]
Limited staffing capacity and need for staff training	[27]		[28,32,33,37,41,43]
Improving active involvement of service users and carers in care decisions	[28,33]	[33]	[28,33,42]

### Methodological quality

Two authors (VP, RA) independently appraised the quality of included studies using the Critical Appraisal Skills Programme (CASP) checklist [24]. The CASP checklist is a structured method for evaluating the quality, credibility and relevance of qualitative research, in which studies are rated on a 10-point score (1 = poor quality, 10 = high quality) based on meeting all 10 CASP criteria. Disagreements were resolved by consensus and discussion with a third author (ES). As appraisal of quality in qualitative research remains a contested area of debate [25], lower scoring studies were not excluded from the review. A sensitivity analysis was undertaken by removing the three lowest quality scoring studies, to assess the influence on synthesised themes.

## Results

**Fig 1:**
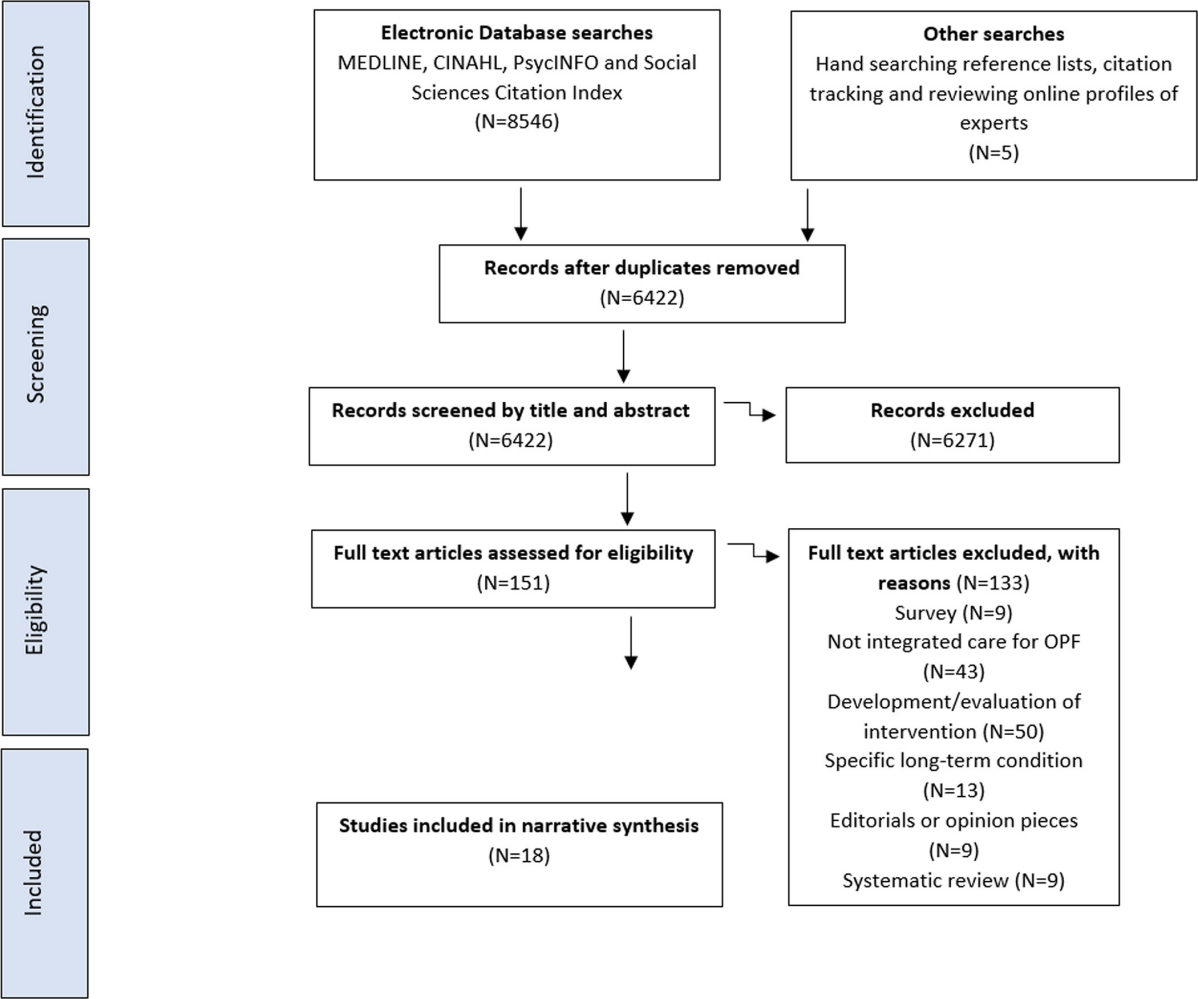
PRISMA diagram of flow of studies through stages of the review.

Database searching identified 8546 articles and following deduplication this yielded 6442 papers. A total of 6,271 articles were excluded on title and abstract screening because they did not meet the inclusion criteria. 133 studies were excluded on full text screening, leaving 13 eligible studies. A further five relevant studies were identified through hand-searching of reference lists and citation tracking of included studies, and reviewing online profiles of experts in the field, resulting in a final total of 18 studies included in this review (Fig 1).

### Study characteristics

Study characteristics are summarised in Table 1. Studies were published between 2009 and 2017. Five studies were from the United Kingdom (UK) [26,27,28,29,30], four from Canada [31,32,33,34], three from the Netherlands [35,36,37], two from Sweden [38, 39] two from the United States [40,41], one from Germany [42], and one was a cross-cultural study (Canada/France) [43]. Most studies (N = 14) examined service user, carer and/or provider perspectives of specific models of integrated care, including: case management [30,35,38], care coordination [31,40,43], transitional care [28,34], continuum of care [39], comprehensive care [42], interdisciplinary primary care models [37,41], or were described generally as an integrated health and/or social care model [26,27]. In addition, one UK study focused on developing an integrated care intervention [29], and three North American studies examined perspectives on how existing services could be better integrated in primary/community care [33, 41], or across different care sectors [32]. Nine studies focused on integrated care interventions or services explicitly targeting OPF [28,29,31,32,37,39,40,41,43], whilst eight studies delivered integrated care to older adults with complex health and social care needs and multimorbidity (with frailty implied) [26,27,30,33,34,36,38,42]. One study comprised of a mixture of older adults with frailty, complex care needs and ‘robust’ participants (without frailty and complex care needs) [35]. Nine studies used interviews and/or focus groups [28,32,33,35,39,40,41,42,43] or a combination of observations and interviews/focus groups [36,38] and analysis of key documents [30]; whilst one study used video stories [26] and five studies used mixed methods designs [27,29,31,34,37].

Four studies [27,35,38,40] examined service user perspectives, one study [26] on the views of service users and carers, whilst six studies [31,36,39,41,42,43] examined the perspectives of professionals only, including physicians, nurses, allied health professionals, geriatricians, social care professionals and health care managers. Seven studies [28,29,30,32,33,34,37] investigated service user/carer and professional views.

### Methodological quality of studies

Overall, the quality of included studies was rated as moderate to high, with CASP scores ranging from 5.5 to 10 (mean score 7.5) (see Table 1). The most common reasons for lower score allocations were poor reporting of potential researcher bias and recruitment strategies. Seven studies did not specify using a theoretical approach [27,29,30,31,32,33,34]. Four studies drew on one or more theoretical perspectives to inform the data collection, analysis and/or interpretation of findings, including sociological [26,39], behavioural and implementation frameworks [42], and organisational psychology theories [36]. Eight studies used methodological frameworks to inform data analysis, including a framework for ethnographic analysis [38], realist [42] and grounded theory approaches [35,40,41,43], framework analysis [28], and a process evaluation model [37]. A sensitivity analysis on the identified themes from included studies suggested that the findings were robust when the three lowest scoring studies [27,34,41] were removed.

## Narrative synthesis

Emerging themes in this synthesis will be discussed in relation to service user, carer and provider: 1) perspectives on integrated care for OPF; and 2) perceptions of factors facilitating and hindering implementation.

### Service user, carer and provider perspectives on integrated care for older people with frailty

We found four themes and related subthemes pertaining to stakeholder perspectives on integrated care for OPF (Table 2).

#### Different preferences for integrated care among service users

Three studies found that service users reported different preferences for integrated care, shaped by differences in perceptions of individual responsibility to manage their health and care needs [26, 35,38]. In one UK study, several service users felt empowered after receiving more services, welcoming opportunities to learn new skills or coping mechanisms and receive self-management support [26]. However, other service users in the same study felt disempowered by an increase in provision of services, which was exacerbated for some by overprotective carers.

In a Swedish study several service users declined the support of a case manager preferring to manage independently with the support of family members [38]. Others felt that they did not have a current need for a case manager but recognised that they may require professional support in the future [38]. In a Dutch study [35] increased provision of services was perceived by service users to enhance their sense of control over their care and lives helping them to manage fears of dependency and anticipated health losses. For example, one service user commented:

*I find it a great reassurance that she [case manager] says ‘We’re here if you need us’* [35].

### System and service organisation components of integrated care for older people with frailty

#### Improved continuity and coordination of care, and multidisciplinary team working

In a number of studies, service users, carers and providers perceived that improved continuity and/or coordination of care were key features of integrated care for OPF [27,28,29,30,32,33,34,35,36,38,40,41]. In six studies [29,30,34,35,38,40] service users and carers valued care coordinator or case manager roles as part of integrated care programmes to improve experiences of continuity of care. In a UK study, both lay and professional stakeholders emphasised the importance of trained key workers to facilitate continuity and coordination of care to improve management of health and social care needs [29]. Service users and carers placed importance on continuity of care through these one-to-one relationships with a care coordinator [40], case manager [30,35,38] or community key worker [29]. In two studies, service users valued such one-to-one roles with professionals to improve the transition of care from hospital to community settings [30], provide appropriate information and support [40], and facilitate personalised care [30].

However, some service users or carers in four studies [27,28,29,33], and providers in two of these studies [29,33] reported a lack of continuity and coordination of care within existing services. Reasons for this included: perceived limited service user and carer involvement during transitions of care between hospital and community settings [28,33] or in the design of integrated care services [33]; inconsistent service delivery between care providers [33]; and organisational factors such as staff shortages and high staff turnover, particularly among care workers [27]. For example, one service user of an integrated care service in a UK study expressed dissatisfaction with poorly coordinated social care, rather than health care, and the negative consequences of this:

*Don’t get the same person [from the care agency] coming around twice*. *That’s the trouble*. *You have got to explain everything each time* ([27], p.504).

Similarly, continuity of care was not perceived as usual practice for OPF in another UK study, as one volunteer carer commented:

*I think older people are very much aware of continuity*. *Old age doesn’t like too much change and that’s where a lot of care falls down*. *The continuity is simply not there* ([29], p.869).

In five studies care providers perceived integrated care for OPF involved improved coordination of services between different providers to deliver comprehensive, holistic care tailored to meet individual needs [33,36,41,42,43]. For example, one health care provider in a German study highlighted:

*Basically it’s a whole network of staff from different professional groups who are linked to one another and who communicate so that the patient is cared for in an optimal way* [42].

In several studies some providers perceived effective MDT working as part of integrated care for OPF included enhancing the capacity of the MDT [28,33,36], integrating social workers in primary care teams [41], and fostering closer collaborative working between primary care physicians (PCPs), geriatricians and case managers [43]. In one Canadian study providers suggested expanding and diversifying the composition of MDT members [33] as a strategy for optimising the delivery of integrated care for this patient group. This viewpoint which was also shared by some service users and carers in the same study, who considered this would reduce PCP workloads:

*So if somehow the ordinary doc who’s working on his own or with one or two other people*, *could get some funding for a nurse practitioner*, *and if the nurse practitioners were available*, *that would be a great first line of defence* [33].

It was also suggested this coordination of services needs to span different care sectors and organisational boundaries [28,30,32,36]. For example, in one Canadian study [32], some providers highlighted the importance of reducing communication points in the system and ‘duplication burden’ for service users and providers.

#### Improved access and navigation of the health and care system for service users and carers

Lay and professional stakeholders in three studies suggested poor system navigation limited care coordination across different provider organisations and care sectors [29,32,33]. In four studies service users, carers, and/or providers attributed improved support to navigate the system to care coordinator [34,40] or case manager [30,38] roles. For example, in one Canadian study [34], all three stakeholder groups reported positive perceptions of intensive geriatric service workers (IGSW), who aimed to improve transitions of care, system navigation and access to follow-up community services, as one provider highlighted:

*It’s a very complicated system that we live in and for the IGSW to go in there and simplify it for them really helps* ([34], p.158).

### Relational aspects of care and support as part of integrated care for older people with frailty

#### Quality and nature of service user-provider relationships

In several studies service users [27,30,35,37,38] and carers [30] highlighted the quality and nature of the relationship they had with their care provider as a key aspect of integrated care. A positive relationship enhanced experiences of continuity and coordination of care across care transitions or the health and social care system [30,38], feeling empowered and in control, safe and secure, being provided with timely information and longer-term monitoring [35], and tailoring of care to meet individual needs [27,30,35,37,38]. Service users placed value on one-to-one relationships with a trusted care professional [38,35], who listened to them and treated them with respect [27,37,38], where there was good communication [35], and who facilitated access to health and social care [27,38,40] and advocated on their behalf [30,38]. For example, one woman in a Dutch study spoke about the positive qualities of the relationship she had with her case manager:

*I think she’s a friendly woman*, *and she’s on a level with you rather than looking down at you*, *and that alone is worth a lot* [35].

Poor relationships between service users and health and social care professionals negatively affected integrated care [27,28,33]. In two of these studies [28,33], some service users experienced limited involvement in hospital discharge planning and conflicting information from providers [28,33]. In one UK study, several recipients of an integrated health and social care service [27] highlighted a lack of perceived interest from their social care provider in establishing genuine rapport with them. For example, one service user commented:

*Agency carers don’t get to know you*. *They didn’t have the right attitude*. *They do the job for money* ([27] p.503).

#### Access to appropriate and timely carer support

In two studies carers experienced improved access to appropriate and timely support as part of integrated care provision [30,34], including enhanced psychosocial support [30], continuity of care and support to navigate and access community services [30,34]. In one Canadian study some family carers spoke about the positive support their relative had received from care co-ordinators during transitions of care from hospital to community services, as well as improved access to respite care services [34]. For example, one female spouse carer in this Canadian study commented on how this had alleviated the burden of care looking after her disabled husband:

*Everything is different*. *We have hope*. *I feel more confident that I can take care of my husband*. *I was lost before and so stressed*. *Now we have lots of help and I’m feeling so much better* ([34], p.157).

In two studies service users, carers and professionals recognised the need to provide more effective support and reassurance for carers within existing integrated care services for OPF [29,32]. For instance, in one UK study different stakeholder groups emphasised the importance of identifying carer needs and supporting their well-being as part of integrated care, especially when care needs of the OPF increased towards the end of their life. For instance, one community nurse said:

*Respite for carers if needed is of prime importance*. *Supporting carers*, *as without them the patient would not be able to stay in their home if their wishes are to stay and end their life [die] at home* ([29], p.867).

### Stakeholder perceptions of outcomes of integrated care for older people with frailty

#### Improved service user and carer outcomes

In several studies service users highlighted improved patient-level outcomes of integrated care [26,27,30,35,40]. Such outcomes included: improved physical functioning [27,30] timely provision of home adaptations [27], quicker perceived recovery from illness due to prompt provision of healthcare [27], improved independence or likelihood of staying in one’s own home [40] and better quality of life [30,40]. Service users in several of these studies also reported improvements in psychosocial outcomes as a result of receiving integrated care, including: reduced social isolation and depression [27], improved choice and control over the care provided [27,35], feeling empowered through learning new skills and knowledge to self-manage chronic health conditions [26], and being kept informed [27]. For example, one service user in a UK study commented:

*I think I was happy about it because [a member of staff] and me sort of kept in touch as to what was going on*. *Yes*, *she told me all about the procedures*. *And why it takes so long*, *you know* ([27], p.501).

#### Improved system and organisational processes and service outcomes

Improved experiences of continuity of care were reported among service users [30,34,35,38,40], carers [30,34], and professionals (case managers) [30]. For example, one service user and carer respectively in the latter UK study commented on the significance of the relationship and support they had with their case manager:

*She’s at the end of that telephone line and all you’ve got to do is pick it up*, *and if she’s off duty then her phone goes to somebody else of equal capability*, *and they will look after you the same way* ([30], p.93).

*I really felt that she was my friend*, *she certainly acted that way*, *I mean her chief concern was the patient but I also felt she was on my side*. *That sounds silly but it’s true* ([30], p.93).

Service users [30,38,40], and carers and providers (30) also voiced improved perceived or expected experiences of coordination of health and social care services as a result of integrated care, including perceived improvements in timely provision of services such as psychosocial [30] and self-management support [32,33,34], and personalised care [30], with the latter viewpoint also shared by carers in the same UK study [30]. Furthermore, all stakeholder groups [30,34,38,40] reported improved access to health and/or social care as a result of integrated care provision, whereas service users [28,34,38,40], and carers [34] and providers [34] to a lesser extent, also spoke about improved support to navigate care transitions across hospital and community services or between community care services.

Furthermore, in nine studies a range of care providers highlighted a number of other organisational and system level improvements as outcomes of integrated care for OPF. These included perceived or expected improved MDT or inter-professional collaboration [33,36,37,41,42], communication [28,30,32,36], management of follow-up [43], coordination of services [30,32] and service user and carer support to navigate the health and care system [32]. Some providers in one US study also perceived improved provision of holistic care through integrating social care professionals into primary care teams [41], whereas other providers in a study in Germany reported improvements in quality of comprehensive geriatric assessments and reduced adverse events through greater involvement of family carers [42].

Providers also highlighted improved perceived service outcomes resulting from integrated care programmes for OPF [30,34]. Specifically, providers in a Canadian study reported a potential reduction in emergency department attendances, hospital admissions and care home placements following a care coordination model to improve transitions of care [34]. This was supported by a UK study which highlighted perceived reductions in hospital admissions as a result of case management integrated care interventions for this patient population [30]. Conversely, several PCPs in a US study perceived a potential negative unintended consequence of higher staff workloads following integration of social workers in primary care due to increased communication demands between providers [41]. For example, one PCP commented:

*I think what we have in mind when we send the patient to a social worker is there will not be a lot of interchange*… *I would prefer the e-mail route rather than very long*, *winding conversations* ([41], p.590).

In summary, we found that similarities and differences in service user, carer and provider perspectives on integrated care for OPF were evident across included studies. Whereas service users and carers more commonly highlighted continuity of care with a professional they could trust and other relational aspects of care as important aspects of integrated care, providers more often emphasised improved coordination of services between providers working in different care sectors and organisational practices to improve MDT working, inter-professional collaboration and communication as strategies for integrating care for this patient group. Different stakeholder groups also highlighted improved support to access and navigate the health and care system as important components and outcomes of integrated care for OPF.

### Service user, carer and provider perceptions of factors facilitating and hindering implementation of integrated care for older people with frailty

Three themes and related subthemes pertaining to service user, carer and provider perceptions of factors facilitating and hindering implementation of integrated care for OPF were evident (Table 3).

### Perceptions of the integrated care intervention and target population

#### Provider views of the perceived value of the intervention

In four studies providers’ perceptions of the integrated care intervention was seen to either hinder or facilitate its implementation (28,31,37,39). In one Canadian study several PCPs who reported high expectations or a lack of information about the intervention limited their participation in integrated health services networks (IHSNs) to facilitate the implementation of integrated care for this patient group [31]. Conversely, other PCPs in the same study perceived that high proportions of older people and specifically targeting frail populations who were perceived to benefit the most, improved their participation in IHSNs to facilitate implementation [31]. For example, one PCP said:

*At the start*, *patients had to be selected based on which ones stood to benefit and which ones didn't (*…*) I fully agreed with the selection*, *and the project needed frail elderly patients* [31].

In a Dutch study of a nurse-led interdisciplinary integrated care model for OPF, providers also felt that intervention complexity acted as a barrier to its implementation in primary care settings [37]. Conversely, providers in three other studies spoke about other factors perceived to facilitate the implementation of integrated care for this patient group [28,37,39]. Such factors included: having rigorous screening criteria for eligibility [37]; ensuring the intervention addressed everyday problems using ‘bottom up’ approaches [39]; and delivering ‘vertically’ integrated healthcare systems [28]. For example, in relation to the latter one provider in this UK study commented:

*The very fact that we’re an integrated organisation*, *so we’ve got community hospitals as part of [the system]*, *is in its nature*, *a really positive step forward so we haven’t got this separation between the acute episode of the pathway of care and then the rehabilitation/onward method of care* [28].

However, in one Swedish study variations in provider perceptions of factors facilitating or hindering integrated care for OPF to improve transitions of care across hospital, primary and community care settings were evident [39]. Some care providers considered the extent to which the intervention could be flexibly tailored to the care context to facilitate implementation. For example, one frontline care provider in this study commented, ‘*It’s all about being flexible and finding solutions together*.’ [39]. In contrast, other providers spoke about a lack of clear information about the intervention as a key factor hindering its implementation.

#### Complexity of care needs of the patient population

In several studies OPF and/or carers highlighted the challenges of managing multiple health conditions which required complex care management [29,32,33,35]. For example, one carer in a UK study spoke about the unpredictability of living with frailty:

*You’ve only got to trip up and feel tired and then you don’t make your meal that evening and then you become physically less able to do things*. *And it’s just progressive*. *Just one trip will*, *or one anything*, *will set the whole of that off*, *and it is the downward spiral* ([29], p.869).

Service users in a Canadian study placed value on holistic care to meet their multiple needs and working with professionals *‘who took the time to see them as a person’* [33]. However, in another Canadian study several service users [32] voiced difficulties they had experienced discussing the complexity of their health conditions and related care needs with health care professionals due to time restraints for consultations.

In two studies some care providers also reported the challenges of implementing integrated care for OPF due to the perceived complexity of care needs of this patient population [29,32]. In one Canadian study [32] several providers felt that this placed increased burden of care on OPF because they commonly experienced multiple assessments by different care providers [32]. Furthermore, in one UK study a number of providers [29] reported that the complex symptom burden among OPF with end of life care needs was perceived to be a challenge to implementing a community-based integrated palliative and supportive care intervention, with several providers recommending targeting patients with complex physical, mental and emotional symptoms [29].

### Service organisational factors influencing implementation

#### Poor communication and the nature of collaborative working practices between providers

In several studies health care providers reported that poor communication and limited collaborative working between providers in different care settings hindered implementation of integrated care for OPF [30,33,36]. In one Canadian study, several service users, carers and health care providers similarly shared the viewpoint that poor or lack of communication between providers working across a range of community-based primary healthcare services hindered implementation of continuity of care for this patient group [33]. Service users and carers in the same study felt that this led to unnecessary repeat assessments and often being asked the same questions over again by different professionals, which was perceived as frustrating [33]. Several carers also reported feeling excluded from important conversations about their relative’s health care after discharge from hospital.

Furthermore, in seven studies providers highlighted that poor understanding, knowledge and clarity of different provider roles and organisations, and differences in working cultures between providers in different care settings hindered implementation of integrated care for OPF [28,30,31,33,36,39,43]. For example, a community hospital provider in one UK study commented:

*We have joined together as a [system] and I think there doesn’t appear to be–they would probably say the same thing about us–our understanding of what the acute does and their understanding of what we [community] do*, *doesn’t seem to be at the moment*, *as good as it could be* [28].

Across six of these studies providers spoke about the importance of improving inter-professional collaborative working practices between providers working in different care settings as a strategy to facilitate implementation of integrated care for OPF [28,31,33,36,39,43]. Such practices were perceived to enable implementation by fostering mutual trust [36], improve communication between different professionals [28,30,36,40], improve knowledge of different working cultures and clarity of professional roles [28,36,39,43] and quality of care across transitions of care [28].

#### Level of engagement of managers, frontline staff and primary care physicians in the implementation process

In one Swedish study several frontline staff and health and social care managers perceived that improved engagement from managers facilitated implementation of integrated care for OPF because it triggered organisational change, the provision of extra resources, and helped to define clearer health and social care professional roles [39]. For instance, one manager spoke about the importance of top down decisions by upper management driving successful implementation:

*This has to be a decision at the top level of the authority*. *I think*. *Maybe even the politicians have to participate and lead the way here*: *‘This is how we are supposed to work’* [39].

In a minority of studies health care providers [32,39] reported the need for frontline clinical staff to be more involved in the planning and developing of integrated care programmes for OPF to facilitate implementation. In one Dutch study several health and social care professionals’ experiences of delivering and implementing community-based integrated care were hindered by their perceived limited influence over care policies and expressed the need for more opportunities to feedback clinical problems to higher management [36]. This viewpoint was also shared among several care providers and managers in one Swedish study implementing a continuum of care programme for OPF across hospital, primary and community care settings [39]. In addition, in another Canadian study a number of frontline staff and other providers similarly viewed this as a key challenge to implementing effective system integration for this patient population across different care settings [32].

Furthermore, in several studies perceptions of limited involvement of PCPs among providers was viewed as a barrier to implementing integrated care for OPF [30,31,39]. Perceived factors hindering implementation among PCPs included time pressures [39,41]; lack of clarity of roles and skills of other professionals involved in integrated care provision such as social workers [41], case managers and other non-medical staff [43]; lack of information and perceived limited impact of the intervention [31]; and poor existing collaborative working practices and negative relationships between geriatricians and PCPs or doctor/patient relationships [31]. Conversely factors perceived to facilitate implementation of integrated care for OPF among PCPs included: positive collaborative working practices between providers and developing close working relationships between PCPs and case managers [31,43], geriatricians [43], practice nurses and social workers [41]. For example, a PCP delivering an integrated care model for OPF to improve care coordination and collaboration in France as part of a French/Canadian cross-cultural study spoke positively about the role of the case manager (CM), pointing out:

*The CM does what I don’t have time to do*, *i*.*e*. *organizing the environment*, *like contacting nurses*, *family members and assistants*, *giving shape to this process*, *making appointments*, *contacting ambulance services*, *etc*. *This is an unending chore*. *It’s also a big benefit to know that these things are going to get done* ([43], p.319).

### System level factors influencing implementation

#### Limited support for service users and carers to navigate and access the health and care system and availability of infrastructure to support and fund integrated care

In several studies different stakeholder groups perceived that system-level factors related to limited support for service users and carers to navigate and access the health and care system were major barriers to implementation of integrated care for OPF [28,29,32,33,42]. In two Canadian studies, both lay and professional stakeholder groups considered this was related to service users not having the right information to contact the correct services, especially among those without a family carer or where English was not the first language [32,33]. In one of these studies, poor system navigation was also evident among service user, carer and provider accounts of community-based primary care services [33], which was considered a major barrier to implementing continuity of care and system integration of services for this patient population. Several service users and carers in this study experienced the healthcare system as confusing and complex, and felt overwhelmed by the high number of different providers involved in their care which led to difficulties keeping track of the care being provided. For example, one service user commented:

*It was just keeping track of it all*. *It would have been nice to have just one [phone number] but you had to have all different phone numbers*, *all different people* [33].

In the same study, some service users proposed that one solution to improve system navigation to facilitate implementation of integrated care focused on using patient advocates during transitions of care following discharge from hospital and to optimise opportunities for active participation in decisions about their care, especially for those without carers. For example, one service user said:

*I would like to see a position of a patient advocate in the hospitals…and they would know*, *you know*, *when they should be looking at different types of care or know what the situation is at the home*. *And especially if there’s no family or anybody–if they’re–if they don’t have anybody to speak for them*, *I think that would be a really–that’s what I would like to see* [33].

In several studies service users and carers highlighted problems of reduced access to health and care services as a key factor hindering implementation of integrated care [29,32,33]. Some service users in one Canadian study [33] spoke about their experiences of limited access to holistic care, which they felt was related to funding limitations, as well as the lack of transport in rural areas. Several providers in another Canadian study also reported perceived limited access to respite services for carers as a barrier to implementing integrated care for OPF [32].

In four studies a number of care providers [28,32,33,42] perceived that access to shared information technology (IT) systems facilitated implementation of integrated care for OPF because this fostered MDT cooperation and collaborative working between providers to improve coordination of care for this patient population. However, some providers in two Canadian studies [32,33] and one UK study [28] spoke about the lack of shared access to IT systems among professionals working in different care sectors hindering communication between different providers and coordination of health and social care services [28,32]. Several providers in one German study also reported existing fragmented patient information systems hindered MDT collaboration to implement effective geriatric comprehensive care as part of integrated care provision for this patient group, leading to higher perceived staff workloads and administration tasks [42]. In addition, some providers in another Canadian study proposed the development of shared standardised assessments and care pathways would improve the consistency and quality of integrated care implemented between different providers working across community-based primary care services [33].

Furthermore, in a few studies several providers felt that restrictions in funding reimbursement systems for healthcare hindered the implementation of holistic care tailored to the individual needs of OPF [33,42]. In one German study health care professionals highlighted inflexible reimbursement systems influencing the delivery of standard packages of care which limited the ability of MDTs to deliver flexible integrated care approaches to older populations with complex care needs [42]. This was perceived among some clinicians in the same study to subsequently shape both over and under use of services. In a second Canadian study, PCPs similarly reported limitations in funding reimbursements discouraged them from focusing on preventative care measures, conducting home visits and making referrals to ‘non-essential’ services as part of integrated care [33].

#### Limited staffing capacity and need for staff training

Care providers in two studies [28,32] and service users in one study [27] perceived that limitations in staffing capacity hindered implementation of integrated care. Several providers considered this was related to a shortage of specialists in geriatric medicine and psychiatry [32], reduced staffing in community care settings [28], or reduced social care capacity contributing to delays in hospital discharges and hindering transitions of care [28]. Furthermore, in one UK study some service users perceived that staff shortages and high staff turnover, particularly in social care, hindered continuity of care [27], as one participant pointed out:

*I had a good carer first*. *After she left I had 25 carers within nine months*. *It was nine months of unreliability* ([27], p.504).

In several studies health care professionals highlighted a lack of staff training opportunities hindering implementation of integrated care for OPF [32,33,37,41,43]. In one Canadian study this included a perceived lack of geriatric knowledge and training, as well as training in interprofessional approaches for most health care providers [32]. Similarly, PCPs in a Canadian/French cross-cultural study reported that a perceived lack of training in interdisciplinary collaboration and management of follow-up was a key barrier to implementing a care coordination model to improve integrated health and social care provision for this patient population [43]. In another US study several PCPs also spoke about the need for training in managing psychosocial issues and clearer information on the skills and training of social workers to improve integrated care working for OPF in primary care settings [41]. Furthermore, some PCPs in another Canadian study reported the need for training to improve their understanding of different provider roles and how their professional role fitted within the wider MDT, in order to improve continuity of care and system integration in community-based primary health care services for older populations with complex care needs [33]. In addition, in one Dutch study a number of PCPs and other health care providers identified several factors perceived to facilitate the implementation of integrated care for OPF, including improved on the job training and opportunities to exchange experiences with other members of the MDT [37].

#### Improving active involvement of service users and carers in care decisions

In a few studies several health care providers perceived that limited involvement of service users and carers in care decisions hindered the implementation of effective transitions of care between hospital and community care settings for OPF [28,33]. For example, in one UK study several health care professionals spoke about their commitment to involving patients and family members in the hospital discharge planning and process, whilst others recognised that time restraints often limited their involvement during this transition of care:

*Patients and families should be involved in every discharge or transfer because that’s our policy* [28].

*I think sometimes patients have good discharge planning*, *where they know exactly what’s happening*, *however if there is a shortage of beds*, *I think it’s up and out*, *that’s my impression* [28].

In two studies a number of different stakeholder groups perceived that active involvement of family carers in discussions and decisions about the older care recipient’s care enhanced the quality of integrated care provided [33,42]. In one German study, several health care professionals felt that actively involving family members in care decisions, improved the quality of care provided in hospital as part of a MDT integrated geriatric care model and reduced potential adverse clinical events [42]. Furthermore, in another Canadian study [33] both lay and professional stakeholders considered involving family carers enhanced continuity of care and system integration in community-based primary care services for this patient population.

In summary, we identified similarities and differences in service user, carer and provider perceptions of factors facilitating and hindering implementation of integrated care for OPF. Different stakeholder groups perceived the complexity of care needs of this patient population, difficulties with system navigation and access, and limited service user and carer involvement in care decisions as factors hindering implementation. Providers also mainly highlighted a range of other organisational and system factors perceived to facilitate or hinder implementation of integrated care for OPF.

## Discussion

We identified 18 qualitative studies from which we synthesised four themes related to stakeholder perspectives on integrated care for OPF, and three themes related to stakeholder perceptions of factors facilitating and hindering implementation. To the best of our knowledge this is the first systematic review and narrative synthesis that has synthesised the views on three separate stakeholder groups on how integrated care for OPF is perceived to work and how it is implemented.

We found similarities and differences in stakeholder perspectives on integrated care for OPF. The first theme, *different preferences for integrated care among service users* indicated different attitudes towards integrated care among OPF, shaped by different perceptions of individual responsibility and agency influencing how OPF manage their health and care needs.

Second, *system and service organisation components of integrated care for OPF* were highlighted among both lay and professional stakeholders as important aspects of integrated care for this patient population, with improving access and support to navigate the health and care system identified as key aspects. However, whereas service users and carers particularly valued continuity of care with a professional they could trust, providers placed importance on improved coordination of care between providers in different care settings as strategies for integrated care.

Third, the theme *relational aspects of care and support as part of integrated care for OPF* found that service users and carers, rather than providers, placed value on relational aspects of integrated care in terms of the nature and quality of service user-provider relationships and availability of appropriate carer support. Finally, *stakeholder perceptions of outcomes of integrated care for OPF* highlighted the shared importance among different stakeholder groups of improving system and organisational-level outcomes, but also differences among stakeholders, with service users also placing value on improved individual-level outcomes and all stakeholder groups emphasising improved organisational processes for integrated care. Despite key national and international policy drives for integrated care [44,45], service outcomes were less commonly reported among professional and lay stakeholders as outcomes of integrated care for OPF.

Similarities and differences in stakeholder perceptions of factors facilitating and hindering implementation of integrated care for OPF were also evident. First, in terms of the theme *perceptions of the integrated care intervention and target population* both lay and professional stakeholders reported the complexity of care needs of OPF as a barrier to implementation. Providers also reported variations in the perceived value and benefits of the integrated care intervention. Second, *service organisational factors influencing implementation* mainly reported by providers reflected poor communication and the nature of collaborative working practices between providers, as well as level of engagement of managers, frontline staff and PCPs in the implementation process.

Finally, *system level factors influencing implementation* were largely reported by providers, and to a lesser extent service users and carers. These system level factors included limited support for service users and carers to navigate and access the health and care system and availability of infrastructure to support and fund integrated care, limited staffing capacity, training and involvement of service users and carers in care decisions.

### How our findings relate to the existing literature

Our findings highlight the multidimensional ways in which integrated care for OPF was understood from the perspectives of service users, carers and providers. The literature currently indicates uncertainty about which models and combinations of components of integrated care are most effective in improving outcomes for OPF [16] and complex needs [17]. In their recent publication, Briggs and colleagues [46] found that the most commonly reported components of integrated care for older people target clinical level strategies with a paucity of information and evidence on organisational and system level strategies. Our synthesis contributes to addressing this gap in the literature by highlighting that organisational and system level strategies were viewed among different stakeholder groups as key components and outcomes of integrated care for OPF. Notably lay and professional stakeholders similarly placed importance on strategies designed to improve access and support to navigate the health and care system. However, whereas service users and carers emphasised ‘relational continuity of care’ as part of an ongoing one-to-one relationship with a provider they could trust, providers more commonly spoke about ‘management continuity of care’ focusing on improved coordination of care and services across different organisational boundaries [47] as strategies for integrated care for this patient population. OPF and their carers also placed importance on other relational aspects as part of integrated care, namely quality of relationships with care providers and access to carer support, which corroborates a limited number of other qualitative studies [48,49,50].

Findings from our review also highlight that multiple level contextual factors were perceived among stakeholders to facilitate and hinder implementation of integrated care for OPF, which concurs with other reviews examining factors influencing integrated care interventions for older populations [19,20,51]. Organisational and system level contextual factors were viewed among stakeholders, particularly providers, to play a key role in the successful implementation of models of integrated care for OPF. Similar findings have been reported in a realist review of evaluations of integrated care programmes for older adults with complex needs [20], and a recent Delphi global consensus study to prioritise actions to improve the implementation of integrated care systems for older populations in general in community settings [52]. In terms of the latter, key priority actions required to implement person centred integrated care for older people in community settings, include: the importance of actively engaging older people, their family members and local communities; improving capacity, training and support of the paid and unpaid workforce; establishing effective comprehensive assessments and community based services; policy, governance and funding arrangements; and the technological infrastructure to support integrated care working [52]. In our study, we also found that lay and professional stakeholders perceived the nature and complexity of the target population as a further barrier to implementing integrated care, as well as variations among providers in the perceived value and benefits of the integrated care intervention being delivered.

### Strengths, limitations and robustness of the synthesis

A strength of our systematic review and narrative synthesis is that we employed rigorous methods to search and synthesise evidence from existing qualitative studies. We recognise that it is possible that some potentially relevant papers may have been missed as our search strategy focused only on studies published in the English language, and also excluded potentially relevant studies in the Grey literature. The methodological quality of included studies was appraised using the CASP checklist [24], and study quality overall was found to be moderate to high. A sensitivity analysis of themes which involved removing the three lowest scoring studies indicated that the themes and related subthemes from the synthesis were still robust. We excluded studies which focused on a single long term condition. Therefore, the review findings may not be generalisable to older adults with a single long term condition. Finally, we did not develop a conceptual model as specified in our protocol. On synthesis of the literature we identified several differences and similarities in perspectives across stakeholders, as well as factors which facilitate and hinder implementation. These require further study before a conceptual model for integrated care can be developed.

### Conclusions and implications for policy, practice and research

The findings from this review have a number of implications for policy, practice and research focusing on integrated care for OPF and its implementation. First, we identified different preferences for integrated care among OPF. Future integrated care interventions and/or quality improvement initiatives should explore a tailored approach to cater for such preferences as part of a person-centred care approach. Second, our findings highlight the perceived importance among stakeholders of organisational and system level components of integrated care for OPF, in particular improving support for access and navigation of the health and care system. Overall, we found support among stakeholders for a model of integrated care underpinned by a continuity of care approach. However, lay and professional stakeholders tended to emphasise different forms of continuity of care. Service users and carers particularly valued relational elements of continuity of care, which included professionals working in care coordinator or case manager roles, addressing the nature and quality of service user-provider relationships and access to appropriate and timely carer support. Providers placed more importance on care coordination elements, focusing on improving coordination of care between providers working in different care settings. Similar differences in understandings of continuity of care have also been found in other studies examining continuity of care for people with long term conditions [47,53]. The issue raised is how best to design and evaluate models of integrated care for OPF that meets both needs. We suggest this can be achieved through co-design approaches, in which greater attention is given in policy, practice and research to establishing effective ways in which service users, carers and providers can collaboratively work together, underpinned by a process of meaningful co-production [54], to co-design, implement and evaluate integrated care programmes for this patient population.

Third, our review highlights the importance of evaluating integrated care programmes for OPF using multidimensional outcomes which capture structural and process outcomes as well as patient level outcomes. Finally, we highlight factors perceived by stakeholders to facilitate implementation included the provision of clearer information about the intervention components and the implementation process, training in complex care needs of this patient population and opportunities for peer practice learning. Factors perceived to hinder implementation of integrated care for OPF included the perceived complexity of the intervention and target population, and the importance of multi-level contextual factors. Proposed strategies to address these perceived barriers include: improving communication and collaborative working practices between providers working in different care sectors and across care boundaries, supported by shared IT systems; improved support for service users and carers to access and navigate the health and care system through the development of care coordinator roles working across different care boundaries and settings; sufficient funding and access to resources; engagement of multiple providers at different levels of the organisation in the implementation process; and training of health and social care professionals in interdisciplinary collaboration and to improve knowledge of different provider roles within the MDT. Novel intervention designs which target the development and evaluation of these strategies to improve the implementation of integrated care for this patient population, for example through effectiveness-implementation hybrid designs [55] are warranted.

## Supporting information

S1 File MEDLINE search strategy. (DOC)

S2 File PRISMA checklist. (DOC)
