# Supplementary material for: Service user, carer and provider perspectives on integrated care for older people with frailty, and factors perceived to facilitate and hinder implementation: A systematic review and narrative synthesis
Source: PLoS One. 2019 May 13;14(5):e0216488. doi: 10.1371/journal.pone.0216488 (PMC6513075; doi:10.1371/journal.pone.0216488)
Supplement: S1 File — (DOC) [file pone.0216488.s001.doc]

**Supporting Information file**

MEDLINE search strategy

((service user* OR (frail elderly) OR old* OR frail OR older adult* OR older people OR older person* OR caregiver* OR carer* OR (health care provider*) OR (health care professional*) OR (health care personnel) OR (general practitioner*) OR GP OR ("physician, family") OR (social care provider*) OR (social worker*) OR nurse* OR (allied health professional*) OR (allied health personnel))

AND ((delivery of health care, integrated) OR ("integrated care pathway*") OR (integrated care) OR (integrated care model*) OR (collaborative care) OR (continuity of care) OR (Continuity of Patient Care) OR (“Buurtzorg model”) OR care coord* OR care co-ord* OR comprehensive care OR (“comprehensive geriatric assessment”) OR (“case manag*”))

AND ((qualitative research) OR (qualitative study) OR perspective* OR view* OR experience* OR ("attitude of health personnel") OR (“attitude of social care personnel”) OR ("health knowledge, attitudes, practice") OR opinion* OR perception* OR implementation OR (health plan implementation) OR barrier* OR hinder* OR enabl* OR facilitat*))
